# Gallstones after bariatric surgery: mechanisms and prophylaxis

**DOI:** 10.3389/fsurg.2025.1506780

**Published:** 2025-03-13

**Authors:** Shenhao Chen, Yamin Zheng, Jie Cai, Yuzhao Wu, Xi Chen

**Affiliations:** ^1^ Department of General Surgery, Xuanwu Hospital, Capital Medical University, Beijing, China; ^2^ The First Clinical Medical College, Xuanwu Hospital, Capital Medical University, Beijing, China; ^3^ Department of Health Management, Xuanwu Hospital, Capital Medical University, Beijing, China

**Keywords:** bariatric surgery, gallstone, Roux-en-Y gastric bypass, sleeve gastrectomy, ursodeoxycholic acid, probiotics

## Abstract

Gallstones represent a common yet often underappreciated complication following bariatric surgery, with reported incidence rates ranging widely from 10.4% to 52.8% within the first postoperative year. Multiple factors contribute to gallstone formation in this setting, including intraoperative injury to the hepatic branch of the vagus nerve, alterations in bile composition, reduced food intake, shifts in gastrointestinal hormone levels, and dysbiosis of the gut microbiota. Notably, the risk of cholelithiasis varies by surgical procedure, with sleeve gastrectomy (SG) generally associated with a lower incidence compared to Roux-en-Y gastric bypass (RYGB). Prophylactic cholecystectomy during bariatric surgery may benefit patients with preexisting gallstones, whereas preserving the hepatic branch of the vagus is an important technical consideration, particularly in RYGB, to mitigate postoperative gallstone risk. Pharmacological interventions, such as ursodeoxycholic acid (UDCA), have demonstrated efficacy in preventing gallstones and reducing subsequent cholecystectomy rates. However, consensus is lacking on the optimal dosing, duration, and administration frequency of UDCA across different bariatric procedures. Additionally, dietary measures, such as moderate fat intake or fish oil supplementation, have shown promise in alleviating lithogenic processes. Emerging evidence supports the use of probiotics as a safe and patient-friendly adjunct or alternative to UDCA, given their ability to improve gut dysbiosis and reduce gallstone formation. Further high-quality studies are needed to define standardized prophylactic strategies that balance efficacy with patient adherence, offering personalized gallstone prevention protocols in the era of widespread bariatric surgery.

## 1 Introduction

Obesity is a chronic, multifactorial disease characterized by excessive adipose tissue accumulation, which can adversely affect health. It is a well-established risk factor for various metabolic and cardiovascular diseases, including type 2 diabetes, hypertension, osteoarthritis, obstructive sleep apnea, and certain malignancies (1). In 2022, an estimated 43% of adults worldwide aged 18 years and older were classified as overweight (BMI ≥ 25 kg/m^2^), with 16% meeting the criteria for obesity (BMI ≥ 30 kg/m^2^) (2). According to the 2022 guidelines from IFSO and ASMBS (3), bariatric surgery is recommended for individuals with a BMI ≥ 35 kg/m^2^, irrespective of obesity-related comorbidities. For individuals with a BMI of 30.0–34.9 kg/m^2^ and metabolic conditions such as type 2 diabetes, hypertension, or obstructive sleep apnea, surgical intervention may be considered. Furthermore, in Asian populations, bariatric surgery is recognized as a viable treatment option for individuals with a BMI of 27.5–32.4 kg/m^2^ and associated metabolic diseases.

Extensive clinical evidence has demonstrated that bariatric surgery is effective in achieving sustained weight loss and improving obesity-related comorbidities in the majority of patients (4). Currently, the most commonly performed bariatric procedures are sleeve gastrectomy (SG) and Roux-en-Y gastric bypass (RYGB). With an increasing number of patients undergoing bariatric surgery, postoperative complications have garnered significant attention. Gallstones are frequently observed following bariatric surgery, with a subset of patients developing symptomatic cholelithiasis necessitating cholecystectomy (5–12). The high incidence, challenges in prevention, and potential impact on postoperative quality of life underscore the clinical significance of gallstone formation in this population. The following review will provide a comprehensive analysis of the underlying mechanisms and prophylactic strategies for cholelithiasis following bariatric surgery.

## 2 Relationship between obesity, bariatric surgery and gallstones

### 2.1 Increased prevalence of gallstones in patients with obesity

Cholelithiasis is a prevalent condition, affecting approximately 10%–20% of the global adult population, with obesity recognized as a significant risk factor for gallstone formation (13). The association between general adiposity, as measured by body mass index (BMI), and gallstone disease has been well established. Epidemiological studies have demonstrated that for every five-unit increase in BMI (kg/m^2^), the hazard ratio for cholelithiasis increases by 31% (14). In addition to abdominal adiposity, central obesity, characterized by visceral fat accumulation, has been identified as a significant contributor to gallstone development. Metrics such as the weight-adjusted waist circumference index (WWI), visceral adiposity index (VAI), and waist-to-hip ratio (WHR) have been independently linked to an increased risk of cholelithiasis. Specifically, each unit increase in WWI is associated with a 34% increase in gallstone prevalence, while each unit increase in VAI and WHR corresponds to a 31% and 18% increase, respectively (14–16). These findings underscore the multifaceted role of adiposity in gallstone pathogenesis and highlight the importance of obesity management in reducing the risk of cholelithiasis.

### 2.2 Elevated risk of gallstones following bariatric surgery

Clinical studies have reported a wide range in the incidence of gallstones (both symptomatic and asymptomatic cases) following bariatric surgery, with rates varying from 10.4% to 52.8% within 6–12 months postoperatively, while the incidence of symptomatic gallstones in patients who did not receive prophylactic gallstone-lowering interventions has been reported to range from 3.0% to 22.9% (5–12, 17, 18). However, these studies are limited by relatively small sample sizes and short follow-up durations. Furthermore, previous research has predominantly focused on the development of symptomatic gallstones after bariatric surgery, while the long-term risks associated with asymptomatic gallstones remain underexplored. Notably, asymptomatic gallstones can progress to serious complications, including cholecystitis, cholangitis, obstructive jaundice, pancreatitis, and, in rare cases, gallbladder carcinoma, highlighting the need for proactive surveillance and management strategies (19). Given the potential clinical consequences, the overall incidence of gallstones following bariatric surgery warrants further investigation and consideration in postoperative care protocols.

### 2.3 Variations in gallstone incidence among different bariatric procedures

Although the incidence of gallstones significantly increases after bariatric surgery, there are still notable differences between surgical procedures. Compared with those receiving RYGB, the summary results showed that participants receiving SG had a 35% lower rate of cholelithiasis, which may be attributed to differences in postoperative weight loss dynamics (20). SG is a restrictive procedure that induces weight loss primarily by reducing gastric volume and limiting caloric intake, whereas RYGB combines restrictive and malabsorptive mechanisms, bypassing a segment of the jejunum to reduce nutrient absorption. Rapid weight loss, exceeding 1.5 kg per week through very-low-calorie diets or following bariatric surgery, has been associated with gallstone formation in up to 30% of individuals (21). Within the first year postoperatively, patients undergoing RYGB experience an average weight loss of approximately 31.2%, compared to 25.2% in those undergoing SG (22). The greater short-term weight reduction associated with RYGB may partly explain the higher incidence of gallstone formation observed in these patients.

## 3 Mechanism of gallstone formation after bariatric surgery

### 3.1 Vagus nerve injury

The vagus nerve innervates multiple organs, particularly within the gastrointestinal tract and associated digestive glands. As it descends along the anterior and posterior surfaces of the esophagus into the abdomen, the anterior vagal trunk bifurcates into the gastric and hepatic branches. The hepatic branch of the vagus nerve plays a crucial role in regulating gallbladder contraction and the relaxation of the Sphincter of Oddi. While bariatric surgery is a well-known risk factor for postoperative gallstone formation, it is not the only surgical intervention associated with this complication. A study by Little et al. (23) reported a 28% incidence of new gallstone formation three years after abdominal surgery. Additionally, gastric cancer surgeries involving vagal nerve disruption have been linked to increased gallstone formation, whereas preservation of the hepatic branch has been shown to reduce the risk of postoperative cholelithiasis (24–26).

Despite the recognized role of the vagus nerve in gallbladder function, no studies to date have specifically quantified the incidence of vagus nerve injury during bariatric surgery. In RYGB, the stomach is transected using a surgical stapler, initially in a horizontal direction, followed by a vertical transection toward the angle of His. However, during the division of the gastrohepatic ligament at the lesser curvature of the stomach, prior to gastric pouch creation, the hepatic branch of the vagus nerve is often neither identified nor isolated by most surgeons (27). This oversight increases the risk of inadvertent injury due to the nerve's anatomical proximity. In contrast, SG involves creating a narrow gastric sleeve by vertically stapling the stomach and excising the greater curvature. The gastrohepatic ligament is preserved during SG, which results in a lower risk of vagus nerve injury compared to RYGB. Consequently, the incidence of gallstones following RYGB is higher than that observed after SG, potentially due to the increased likelihood of hepatic branch injury during the procedure.

### 3.2 Abnormal bile composition

Bile is a viscous, yellow-green digestive fluid composed of water, cholesterol, phospholipids, bile salts, small amounts of proteins, and inorganic salts (21). Gallstone formation following bariatric surgery is primarily attributed to alterations in the chemical composition of bile. Due to reduced food intake, the demand for bile in digestion decreases. However, hepatic bile synthesis remains relatively constant. Consequently, bile stored in the gallbladder becomes excessive and increasingly concentrated.

After RYGB, altered gastrointestinal anatomy leads to reduced intestinal cholesterol absorption, resulting in significantly decreased serum low-density lipoprotein cholesterol levels (28). To compensate, hepatic cholesterol catabolism is upregulated (28). In contrast, SG is a purely restrictive procedure that minimally impacts intestinal cholesterol absorption (28). In individuals with obesity, approximately 50% of cholesterol is stored in peripheral adipose tissue in a free form. Rapid weight loss following bariatric surgery leads to a substantial reduction in adipose tissue mass, resulting in the release of large amounts of free cholesterol (29). This excess cholesterol is secreted into bile, leading to bile supersaturation and subsequent gallstone formation (29).

Collectively, these physiological changes contribute to increased cholesterol concentration in bile following bariatric surgery. Elevated cholesterol levels can impair the contractility of gallbladder smooth muscle cells by altering the plasma membrane, thereby promoting cholestasis and facilitating gallstone formation (30). Furthermore, changes in gallbladder bile composition, particularly during rapid weight loss, have been shown to involve mucin as a significant factor in cholesterol crystal and gallstone formation. Shiffman et al. (31) demonstrated that mucin plays a crucial role in this process. The viscosity of bile is directly correlated with mucin concentration. Mucin can accelerate the precipitation of cholesterol crystals by forming a high-viscosity gel and mucin network, acting as a matrix and scaffold for stone growth (32).

### 3.3 Decreased food intake

Following bariatric surgery, patients experience a significant reduction in food intake due to decreased gastric volume, reduced gastric motility, and diminished appetite. This decreased food consumption weakens the nerve reflex that stimulates gallbladder contraction as food passes through the gastrointestinal tract, leading to cholestasis and facilitating gallstone formation. Johansson et al. (33) reported that the incidence of symptomatic gallstones was three times higher in patients adhering to a 500 kcal per day diet compared to those consuming 1,500 kcal per day. Additionally, post-surgical reductions in food intake and rapid weight loss impair gallbladder emptying, leading to increased residual volume and decreased refilling, which together promote gallbladder stasis and, consequently, the formation of gallstones (34).

### 3.4 Gastrointestinal hormone disorder

Cholecystokinin (CCK), primarily synthesized by I cells in the intestinal mucosa, promotes gallbladder contraction and relaxation of the sphincter of Oddi through binding to cholecystokinin receptors (CCK-R). I cells secrete CCK in response to the presence of fatty acids and amino acids in the duodenum. Zhu et al. (35) found that plasma CCK levels are elevated in patients with gallstones compared to healthy individuals, suggesting that a defect in CCK-R within the gallbladder may impair its motor function. Following bariatric surgery, particularly RYGB, the reconstruction of the gastrointestinal tract and reduced food intake limit contact with I cells. This may lead to a decrease in plasma CCK levels, impairing gallbladder contraction and promoting gallstone formation. Interestingly, however, it has been shown that plasma CCK concentrations increase after bariatric surgery (36). The relationship between gallbladder emptying, CCK, and CCK-R post-bariatric surgery remains underexplored, presenting potential avenues for novel postoperative gallstone therapies. Notably, sleeve gastrectomy (SG) is associated with a more significant increase in CCK compared to RYGB (37), which may explain the lower incidence of gallstones following SG.

Glucagon-like peptides (GLPs) are synthesized by enteroendocrine L-cells in the intestine. GLP-1 promotes insulin secretion and inhibits glucagon secretion, while GLP-2 inhibits intestinal apoptosis and stimulates intestinal villus hyperplasia. Evidence suggests that levels of both GLP-1 and GLP-2 increase after bariatric surgery (36). Following RYGB, the absence of mechanical restriction between the stomach and duodenum allows for the rapid delivery of nutrients to the jejunum and ileum, which stimulates L cells to synthesize and secrete large amounts of GLP-1 (38). Similarly, SG, which significantly reduces gastric volume, increases intragastric pressure and accelerates gastric emptying, effectively creating a functional intestinal bypass (38). This mechanism, similar to that of RYGB, promotes substantial GLP-1 secretion by L cells. Gether et al. (39) demonstrated that activation of GLP-1 and GLP-2 receptors prolongs gallbladder refilling time and increases gallbladder volume, thereby elevating the risk of gallstone formation. However, Sodhi et al. (40) reported that GLP-1 agonists did not increase the risk of biliary disease compared to other weight loss agents unrelated to GLP-1. Furthermore, an animal study found that liraglutide (a GLP-1 analogue) could prevent gallstone formation by increasing bile acid secretion and decreasing the cholesterol saturation index (41). This research suggests that liraglutide may offer a novel approach to treating or preventing gallstones. Therefore, further investigation into the relationship between GLPs and gallstone formation after bariatric surgery is warranted, as this could inform guidelines for the prevention and treatment of postoperative gallstones.

### 3.5 Gut microbiota dysbiosis

Following bariatric surgery, the composition of the gut microbiota is influenced by several factors, including dietary changes, reconstruction of the gastrointestinal tract, alterations in gut pH, modified gastrointestinal transit time, and changes in bile acid metabolism (42). A prominent alteration observed in most studies is a reduction in the relative abundance of *Firmicutes* postsurgery, accompanied by a corresponding increase in *Bacteroidetes* and *Proteobacteria* after SG and RYGB, respectively (43). Similarly, in a mouse model of gallstones, there is a decrease in *Firmicutes* and the *Firmicutes*-to-*Bacteroidetes* ratio, while *Verrucomicrobia* increases significantly (44). Dysbiosis of the gut microbiota may contribute significantly to the pathogenesis of gallstone formation by regulating bile acid metabolism, primarily through bile salt hydrolases, which de-conjugate bile acids, and 7*α*-dehydroxylase, which converts primary bile acids to secondary bile acids (44). Additionally, due to the reduced motility of the gallbladder after surgery, more bile secreted by the liver is diverted to the intestine. This increased bacterial catabolism of bile salts results in higher levels of biliary deoxycholate, which subsequently promotes hepatic cholesterol hypersecretion and cholesterol crystallization, further contributing to gallstone formation (21). Guman et al. (29) also found that patients who developed gallstones after bariatric surgery exhibited a higher abundance of *Ruminococcus*, a microorganism recently identified as a biomarker for gallstone formation. In contrast, patients who did not develop gallstones postoperatively had a higher abundance of *Lactobacillaceae* and *Enterobacteriaceae* in their gut microbiota (29). *Lactobacillus* species, known to produce bile salt hydrolase, facilitate the dissociation of bile acids in the small intestine and participate in bile acid-mediated signaling pathways (45). These findings suggest that *Lactobacillus* may serve as a protective probiotic against gallstone formation following bariatric surgery.

## 4 Prophylaxis for gallstone formation after bariatric surgery

### 4.1 Prophylactic cholecystectomy

Historically, when bariatric procedures were performed via an open approach, concomitant cholecystectomy was routinely conducted in all patients, regardless of gallstone status (46). With the development of laparoscopic techniques in bariatric practice, however, the incidence of performing cholecystectomy at the time of surgery has declined. Currently, there is no clear consensus on whether prophylactic cholecystectomy should be undertaken during bariatric surgery. For obese patients with gallstones in China, concurrent cholecystectomy during bariatric surgery is generally recommended. By contrast, the European Association for the Study of the Liver advises against routine prophylactic cholecystectomy, especially for asymptomatic gallstones (47). Opponents of prophylactic cholecystectomy emphasize that the occurrence of symptomatic gallstones following bariatric surgery is relatively low. They also highlight that adding a cholecystectomy to bariatric surgery prolongs operative time and may elevate surgical risks, given that the gallbladder in obese patients is often enveloped by fatty liver and may necessitate challenging trocar placement.

However, up to 70% of patients with asymptomatic gallstones who undergo laparotomy for unrelated conditions eventually develop biliary symptoms, with 40% requiring cholecystectomy within one year (48). Moreover, laparoscopic cholecystectomy following bariatric surgery can pose significant technical challenges due to altered intraabdominal anatomy, the presence of adhesions, and restricted laparoscopic visualization, all of which may increase the risk of adverse outcomes (49). In addition, following RYGB, the continuity between the stomach and duodenum is disrupted, hindering the advancement of the duodenoscope through the reconstructed Roux limb into the biliopancreatic system. Consequently, endoscopic retrograde cholangiopancreatography (ERCP) and related stone extraction procedures become substantially more complex (50–52). Nevertheless, Allatif et al. (53) have demonstrated that prophylactic cholecystectomy can be safely performed during bariatric surgery by experienced laparoscopic surgeons. In their study, patients undergoing simultaneous bariatric surgery and cholecystectomy did not exhibit a higher rate of minor or major complications compared to those who had bariatric surgery alone. Taken together, these findings suggest that prophylactic cholecystectomy may be advantageous for patients with asymptomatic gallstones undergoing bariatric surgery. For those without gallstones, regular ultrasound monitoring provides an effective approach for timely detection and intervention (54).

### 4.2 Preservation of the vagus nerve in bariatric surgery

RYGB has long been viewed as the gold standard in bariatric surgery due to its superior weight reduction outcomes, although it carries a higher surgical risk (55). In contrast, SG is technically less complex, has a shorter operative duration, and has gained popularity for managing obesity and related comorbidities (55). However, with the increasing number of SG procedures, concerns such as postoperative weight regain and gastroesophageal reflux have become more prominent. Consequently, surgeons and patients must thoroughly weigh the potential risks, benefits, and uncertainties associated with the long-term effects of bariatric procedures (4). A shared decision-making process, guided by patient preferences and values, is therefore recommended. Recent studies indicate that SG is associated with a significantly lower incidence of gallstone disease compared to RYGB (20). Thus, SG may be preferable for patients seeking to minimize postoperative cholelithiasis risk. However, when RYGB is chosen, surgeons must carefully identify or preserve the hepatic branch of the vagus nerve during dissection of the hepatogastric ligament at the lesser curvature of the stomach. Although limited research has addressed vagal preservation in RYGB, evidence from distal gastrectomy for early-stage gastric cancer demonstrates that combining intraoperative nerve monitoring with indocyanine green (ICG)-enhanced fluorescence laparoscopy is both safe and effective, notably reducing the incidence of postoperative gallstones (56). Employing a similar approach in RYGB, using electrophysiological monitoring and fluorescence markers to visualize the vagus nerve, may help avert inadvertent damage to its hepatic branch, thereby reducing gallstone formation postoperatively.

### 4.3 Ursodeoxycholic acid (UDCA)

Prophylactic administration of UDCA can effectively reduce the incidence of *de novo* gallstone formation and subsequent cholecystectomy following bariatric surgery (10, 57, 58). Mechanistically, UDCA inhibits both the intestinal absorption of cholesterol and its hepatic secretion into bile, thereby lowering biliary cholesterol saturation by 40%–60% (59). It also decreases bile acid toxicity, which could otherwise damage cell membranes and promote cholestasis (59). In a meta-analysis, Mulliri et al. (60) provided robust evidence of UDCA's protective effect, demonstrating a significant reduction in the rate of gallstone formation (OR = 0.25, 95% CI: 0.21–0.31, *P* < 0.01), symptomatic gallstone disease (OR = 0.29, 95% CI: 0.20–0.42, *P* < 0.01), and cholecystectomy (OR = 0.33, 95% CI: 0.20–0.55, *P* < 0.01) in patients receiving UDCA prophylaxis compared with controls.

However, the optimal UDCA dose and treatment duration remain to be defined. One study indicated that a daily dose of ≤600 mg led to a significant reduction in gallstone risk with better patient compliance (RR = 0.35; 95% CI: 0.24–0.53; *P* < 0.01), whereas doses >600 mg did not confer a statistically significant benefit (RR = 0.30; 95% CI: 0.09–1.01; *P* = 0.05) (61). In contrast, Magouliotis et al. (49) found that both 500–600 mg and 1,000–1,200 mg daily doses were associated with reduced gallstone formation, and observed no major difference between once-daily 500 mg vs. 250 mg twice daily. Another study similarly reported that 500 mg once daily effectively prevented gallstone disease after SG, whereas 250 mg twice daily was more beneficial following RYGB (5).

Given these varying results, large-scale clinical trials are warranted to determine the optimal dosage, duration, dosing interval, and daily administration frequency of UDCA across different bariatric procedures. Such investigations will help balance the prevention of gallstones with patient tolerance and adherence. Ultimately, individualized treatment strategies that take into account surgical technique, baseline obesity severity, and postoperative weight loss trajectory may further improve patient outcomes.

### 4.4 Diet therapy

A relatively high fat intake, as well as fish oil supplementation, may help prevent gallstone formation in obese patients undergoing rapid weight loss (62, 63). Specifically, consuming 7–10 g of fat daily is recommended to facilitate gallbladder emptying, thus counteracting the lithogenic processes that occur during weight reduction (33, 63). Fish oil, rich in n-3 polyunsaturated fatty acids (PUFAs), alters bile composition by increasing phospholipid species containing eicosapentaenoic and docosahexaenoic acids, while reducing those enriched with linoleic or arachidonic acids (63). Epidemiological data further indicate that dietary PUFAs are inversely associated with gallstone incidence (64, 65). Animal studies support these findings, showing that PUFA intake elevates biliary phospholipid levels and inhibits mucin production, thereby curbing gallstone formation (66). Notably, concomitant administration of PUFA and UDCA appears to dissolve cholesterol gallstones by lowering mucin secretion, decreasing cholesterol saturation, and increasing bile phospholipid and bile acid concentrations, which is a synergy preliminarily confirmed to be safe and effective in a clinical trial (67). Dietary modification after bariatric surgery may thus offer a safer and more patient-acceptable approach to reducing gallstone formation. Moreover, combining PUFA with UDCA potentially allows for a reduced dosage of medication, better therapeutic outcomes, fewer adverse effects, and improved adherence, making it a promising strategy for mitigating gallstone risk in bariatric surgery recipients.

### 4.5 Probiotics treatment

Compared with patients who develop cholelithiasis after bariatric surgery, those who remain gallstone-free exhibit a higher abundance of *Lactobacillaceae* and *Enterobacteriaceae*, suggesting a potentially protective effect against gallstone formation and paving the way for probiotic-based therapies (42). Both animal studies and clinical trials have indicated that probiotics can alleviate intestinal dysbiosis and decrease gallstone risk postoperatively. In a murine model, administration of 1.0 × 10^8^ CFU/g of *Clostridium butyricum* (a prevalent human gut commensal) for five weeks lowered the incidence of gallstones by 43% and displayed notable litholytic effects (68). Similarly, Oh et al. (69) demonstrated in mice that Lactobacillus prevents gallstone formation by reducing hepatic 3-hydroxy-3-methylglutaryl-coenzyme A (HMG-CoA) reductase levels and inhibiting gallbladder mucin expression. Moreover, Han et al. (70) showed that probiotics exerted a gallstone-preventive effect comparable to that of UDCA, with fewer adverse reactions and improved compliance in the probiotics group. Although the precise mechanisms remain under investigation, current data suggest that probiotics may lower serum cholesterol by producing bile salt hydrolase (thus deconjugating bile salts), decreasing deoxycholic acid content in bile through a shorter intestinal transit time, and inhibiting biliary cholesterol secretion (68, 70, 71). Large-scale clinical trials are warranted to further validate the efficacy and safety of various probiotics in preventing postoperative gallstones. Given their favorable tolerability and adherence profiles, probiotics hold significant promise as a safe and effective adjunct therapy for gallstone prevention in patients undergoing bariatric surgery.

## 5 Summary and prospects

Gallstone formation after bariatric surgery appears to be multifactorial, potentially involving intraoperative damage to the hepatic branch of the vagus nerve, changes in bile composition, reduced food intake, altered gastrointestinal hormone levels, and gut microbiota dysbiosis. For obese patients presenting with gallstones, simultaneous cholecystectomy during bariatric surgery may confer additional benefits. SG carries a lower risk of postoperative cholelithiasis, whereas preserving the hepatic branch of the vagus nerve is particularly important during RYGB. Although UDCA has demonstrated efficacy in preventing gallstone formation and subsequent cholecystectomy, further research is needed to determine its optimal dosage, treatment duration, and administration schedule for different bariatric procedures. Moreover, dietary interventions, such as moderate fat intake or fish oil supplementation, warrant additional investigation. Probiotics have shown gallstone-preventive effects comparable to UDCA, with fewer adverse events and higher patient compliance, suggesting a promising therapeutic option for long-term management.

## References

[1] Perdomo CM Cohen RV Sumithran P Clément K Frühbeck G. Contemporary medical, device, and surgical therapies for obesity in adults. Lancet. (2023) 401(10382):1116–30. 10.1016/S0140-6736(22)02403-5 36774932

[2] World Health Organization. Obesity and Overweight. Geneva: World Health Organization (2024). Available online at: https://www.who.int/en/news-room/fact-sheets/detail/obesity-and-overweight (Accessed November 18, 2024).

[3] Eisenberg D Shikora SA Aarts E Aminian A Angrisani L Cohen RV 2022 American society for metabolic and bariatric surgery (ASMBS) and international federation for the surgery of obesity and metabolic disorders (IFSO): indications for metabolic and bariatric surgery. Surg Obes Relat Dis. (2022) 18(12):1345–56. 10.1016/j.soard.2022.08.013 36280539

[4] Arterburn DE Telem DA Kushner RF Courcoulas AP. Benefits and risks of bariatric surgery in adults: a review. JAMA. (2020) 324(9):879–87. 10.1001/jama.2020.12567 32870301

[5] Coupaye M Calabrese D Sami O Msika S Ledoux S. Evaluation of incidence of cholelithiasis after bariatric surgery in subjects treated or not treated with ursodeoxycholic acid. Surg Obes Relat Dis. (2017) 13(4):681–5. 10.1016/j.soard.2016.11.022 28089591

[6] Guzmán HM Sepúlveda M Rosso N San Martin A Guzmán F Guzmán HC. Incidence and risk factors for cholelithiasis after bariatric surgery. Obes Surg. (2019) 29(7):2110–4. 10.1007/s11695-019-03760-4 31001756

[7] Mishra T Lakshmi KK Peddi KK. Prevalence of cholelithiasis and choledocholithiasis in morbidly obese south Indian patients and the further development of biliary calculus disease after sleeve gastrectomy, gastric bypass and mini gastric bypass. Obes Surg. (2016) 26(10):2411–7. 10.1007/s11695-016-2113-4 26910024

[8] Moon RC Teixeira AF DuCoin C Varnadore S Jawad MA. Comparison of cholecystectomy cases after roux-en-Y gastric bypass, sleeve gastrectomy, and gastric banding. Surg Obes Relat Dis. (2014) 10(1):64–8. 10.1016/j.soard.2013.04.019 23932005

[9] Nabil TM Khalil AH Gamal K. Effect of oral ursodeoxycholic acid on cholelithiasis following laparoscopic sleeve gastrectomy for morbid obesity. Surg Obes Relat Dis. (2019) 15(6):827–31. 10.1016/j.soard.2019.03.028 31113752

[10] Sakran N Dar R Assalia A Neeman Z Farraj M Sherf-Dagan S The use of ursolit for gallstone prophylaxis following bariatric surgery: a randomized-controlled trial. Updates Surg. (2020) 72(4):1125–33. 10.1007/s13304-020-00850-2 32666477

[11] Sneineh MA Harel L Elnasasra A Razin H Rotmensh A Moscovici S Increased incidence of symptomatic cholelithiasis after bariatric Roux-en-Y gastric bypass and previous bariatric surgery: a single center experience. Obes Surg. (2020) 30(3):846–50. 10.1007/s11695-019-04366-6 31901127

[12] Talha A Abdelbaki T Farouk A Hasouna E Azzam E Shehata G. Cholelithiasis after bariatric surgery, incidence, and prophylaxis: randomized controlled trial. Surg Endosc. (2020) 34(12):5331–7. 10.1007/s00464-019-07323-7 31858245

[13] Kratzer W Mason RA Kächele V. Prevalence of gallstones in sonographic surveys worldwide. J Clin Ultrasound. (1999) 27(1):1–7. 10.1002/(sici)1097-0096(199901)27:1<1::aid-jcu1>3.0.co;2-h 9888092

[14] Zhang M Bai Y Wang Y Cui H Zhang W Zhang L Independent association of general and central adiposity with risk of gallstone disease: observational and genetic analyses. Front Endocrinol (Lausanne). (2024) 15:1367229. 10.3389/fendo.2024.1367229 38529389 PMC10961427

[15] Zhang G Ding Z Yang J Wang T Tong L Cheng J Higher visceral adiposity index was associated with an elevated prevalence of gallstones and an earlier age at first gallstone surgery in US adults: the results are based on a cross-sectional study. Front Endocrinol (Lausanne). (2023) 14:1189553. 10.3389/fendo.2023.1189553 37396166 PMC10311253

[16] Ke B Sun Y Dai X Gui Y Chen S. Relationship between weight-adjusted waist circumference index and prevalence of gallstones in U.S. Adults: a study based on the NHANES 2017–2020. Front Endocrinol (Lausanne). (2023) 14:1276465. 10.3389/fendo.2023.1276465 37964952 PMC10641849

[17] Iglézias Brandão de Oliveira C Adami Chaim E da Silva BB. Impact of rapid weight reduction on risk of cholelithiasis after bariatric surgery. Obes Surg. (2003) 13(4):625–8. 10.1381/096089203322190862 12935366

[18] Manatsathit W Leelasinjaroen P Al-Hamid H Szpunar S Hawasli A. The incidence of cholelithiasis after sleeve gastrectomy and its association with weight loss: a two-centre retrospective cohort study. Int J Surg. (2016) 30:13–8. 10.1016/j.ijsu.2016.03.060 27063855

[19] Ibrahim M Sarvepalli S Morris-Stiff G Rizk M Bhatt A Walsh RM Gallstones: watch and wait, or intervene? Cleve Clin J Med. (2018) 85(4):323–31. 10.3949/ccjm.85a.17035 29634468

[20] Wan Q Zhao R Chen Y Wang Y Wu Y Wu X. Comparison of the incidence of cholelithiasis after sleeve gastrectomy and Roux-en-Y gastric bypass: a meta-analysis. Surg Obes Relat Dis. (2021) 17(6):1198–205. 10.1016/j.soard.2021.02.003 33785273

[21] Lammert F Gurusamy K Ko CW Miquel JF Méndez-Sánchez N Portincasa P Gallstones. Nat Rev Dis Primers. (2016) 2:16024. 10.1038/nrdp.2016.24 27121416

[22] Arterburn D Wellman R Emiliano A Smith SR Odegaard AO Murali S Comparative effectiveness and safety of bariatric procedures for weight loss: a PCORnet cohort study. Ann Intern Med. (2018) 169(11):741–50. 10.7326/M17-2786 30383139 PMC6652193

[23] Little JM Avramovic J. Gallstone formation after major abdominal surgery. Lancet. (1991) 337(8750):1135–7. 10.1016/0140-6736(91)92796-5 1674022

[24] Chen XJ Li N Huang YD Ren S Liu F Chen L Factors for postoperative gallstone occurrence in patients with gastric cancer: a meta-analysis. Asian Pac J Cancer Prev. (2014) 15(2):877–81. 10.7314/apjcp.2014.15.2.877 24568511

[25] Jiang T Zhang H Yin X Cai Z Zhao Z Mu M The necessity and safety of simultaneous cholecystectomy during gastric surgery for patients with asymptomatic cholelithiasis. Expert Rev Gastroenterol Hepatol. (2023) 17(10):1053–60. 10.1080/17474124.2023.2264782 37795528

[26] Wang CJ Kong SH Park JH Choi JH Park SH Zhu CC Preservation of hepatic branch of the vagus nerve reduces the risk of gallstone formation after gastrectomy. Gastric Cancer. (2021) 24(1):232–44. 10.1007/s10120-020-01106-z 32705445

[27] Ponsky JL Jones N Rodriguez JH Kroh MD Strong AT. Massive biliary dilation after Roux-en-Y gastric bypass: is it ampullary achalasia? J Am Coll Surg. (2017) 224(6):1097–103. 10.1016/j.jamcollsurg.2017.03.004 28344051

[28] Benetti A Del Puppo M Crosignani A Veronelli A Masci E Frigè F Cholesterol metabolism after bariatric surgery in grade 3 obesity: differences between malabsorptive and restrictive procedures. Diabetes Care. (2013) 36(6):1443–7. 10.2337/dc12-1737 23275360 PMC3661782

[29] Guman MSS Hoozemans JB Haal S de Jonge PA Aydin Ö Lappa D Adipose tissue, bile acids, and gut microbiome Species associated with gallstones after bariatric surgery. J Lipid Res. (2022) 63(11):100280. 10.1016/j.jlr.2022.100280 36115596 PMC9672443

[30] Pozo MJ Camello PJ Mawe GM. Chemical mediators of gallbladder dysmotility. Curr Med Chem. (2004) 11(13):1801–12. 10.2174/0929867043364955 15279583

[31] Shiffman ML Sugerman HJ Kellum JM Moore EW. Changes in gallbladder bile composition following gallstone formation and weight reduction. Gastroenterology. (1992) 103(1):214–21. 10.1016/0016-5085(92)91115-k 1612328

[32] Zhao Z Yang Y Wu S Yao D. Role of secretory mucins in the occurrence and development of cholelithiasis. Biomolecules. (2024) 14(6):676. 10.3390/biom14060676 38927079 PMC11201413

[33] Johansson K Sundström J Marcus C Hemmingsson E Neovius M. Risk of symptomatic gallstones and cholecystectomy after a very-low-calorie diet or low-calorie diet in a commercial weight loss program: 1-year matched cohort study. Int J Obes (Lond). (2014) 38(2):279–84. 10.1038/ijo.2013.83 23736359 PMC3921672

[34] Al-Jiffry BO Shaffer EA Saccone GT Downey P Kow L Toouli J. Changes in gallbladder motility and gallstone formation following laparoscopic gastric banding for morbid obestity. Can J Gastroenterol. (2003) 17(2):169–74. 10.1155/2003/392719 12677265

[35] Zhu J Han TQ Chen S Jiang Y Zhang SD. Gallbladder motor function, plasma cholecystokinin and cholecystokinin receptor of gallbladder in cholesterol stone patients. World J Gastroenterol. (2005) 11(11):1685–9. 10.3748/wjg.v11.i11.1685 15786550 PMC4305954

[36] Dimitriadis GK Randeva MS Miras AD. Potential hormone mechanisms of bariatric surgery. Curr Obes Rep. (2017) 6(3):253–65. 10.1007/s13679-017-0276-5 28780756 PMC5585994

[37] Peterli R Steinert RE Woelnerhanssen B Peters T Christoffel-Courtin C Gass M Metabolic and hormonal changes after laparoscopic Roux-en-Y gastric bypass and sleeve gastrectomy: a randomized, prospective trial. Obes Surg. (2012) 22(5):740–8. 10.1007/s11695-012-0622-3 22354457 PMC3319900

[38] Akalestou E Miras AD Rutter GA Le Roux CW. Mechanisms of weight loss after obesity surgery. Endocr Rev. (2022) 43(1):19–34. 10.1210/endrev/bnab022 34363458 PMC8755990

[39] Gether IM Nexøe-Larsen C Knop FK. New avenues in the regulation of gallbladder motility-implications for the use of glucagon-like peptide-derived drugs. J Clin Endocrinol Metab. (2019) 104(7):2463–72. 10.1210/jc.2018-01008 30137354

[40] Sodhi M Rezaeianzadeh R Kezouh A Etminan M. Risk of gastrointestinal adverse events associated with glucagon-like peptide-1 receptor agonists for weight loss. JAMA. (2023) 330(18):1795–7. 10.1001/jama.2023.19574 37796527 PMC10557026

[41] Zhang Z Du Z Liu Q Wu T Tang Q Zhang J Glucagon-like peptide 1 analogue prevents cholesterol gallstone formation by modulating intestinal farnesoid X receptor activity. Metab Clin Exp. (2021) 118:154728. 10.1016/j.metabol.2021.154728 33581130

[42] Komorniak N Pawlus J Gaweł K Hawryłkowicz V Stachowska E. Cholelithiasis, gut microbiota and bile acids after bariatric surgery-can cholelithiasis be prevented by modulating the microbiota? A literature review. Nutrients. (2024) 16(15):2551. 10.3390/nu16152551 39125429 PMC11314327

[43] Davies NK O’Sullivan JM Plank LD Murphy R. Altered gut microbiome after bariatric surgery and its association with metabolic benefits: a systematic review. Surg Obes Relat Dis. (2019) 15(4):656–65. 10.1016/j.soard.2019.01.033 30824335

[44] Wang Q Jiao L He C Sun H Cai Q Han T Alteration of gut microbiota in association with cholesterol gallstone formation in mice. BMC Gastroenterol. (2017) 17(1):74. 10.1186/s12876-017-0629-2 28599622 PMC5466737

[45] Alnouti Y. Bile acid sulfation: a pathway of bile acid elimination and detoxification. Toxicol Sci. (2009) 108(2):225–46. 10.1093/toxsci/kfn268 19131563

[46] Wood SG Kumar SB Dewey E Lin MY Carter JT. Safety of concomitant cholecystectomy with laparoscopic sleeve gastrectomy and gastric bypass: a MBSAQIP analysis. Surg Obes Relat Dis. (2019) 15(6):864–70. 10.1016/j.soard.2019.03.004 31060907

[47] European Association for the Study of the Liver (EASL). EASL clinical practice guidelines on the prevention, diagnosis and treatment of gallstones. J Hepatol. (2016) 65(1):146–81. 10.1016/j.jhep.2016.03.005 27085810

[48] Elgohary H El Azawy M Elbanna M Elhossainy H Omar W. Concomitant versus delayed cholecystectomy in bariatric surgery. J Obes. (2021) 2021:9957834. 10.1155/2021/9957834 34234964 PMC8216831

[49] Magouliotis DE Tasiopoulou VS Svokos AA Svokos KA Chatedaki C Sioka E Ursodeoxycholic acid in the prevention of gallstone formation after bariatric surgery: an updated systematic review and meta-analysis. Obes Surg. (2017) 27(11):3021–30. 10.1007/s11695-017-2924-y 28889240

[50] Spadaccini M Giacchetto CM Fiacca M Colombo M Andreozzi M Carrara S Endoscopic biliary drainage in surgically altered anatomy. Diagnostics (Basel). (2023) 13(24):3623. 10.3390/diagnostics13243623 38132207 PMC10742737

[51] Vara-Luiz F Nunes G Pinto-Marques P Oliveira C Mendes I Patita M Endoscopic treatment of bile duct stones after bariatric Roux-en-Y gastric bypass through endoscopic ultrasound-directed transgastric ERCP. Endoscopy. (2023) 55(S 01):E1065–7. 10.1055/a-2161-3450 37734414 PMC10513776

[52] Amstutz S Michel JM Kopp S Egger B. Potential benefits of prophylactic cholecystectomy in patients undergoing bariatric bypass surgery. Obes Surg. (2015) 25(11):2054–60. 10.1007/s11695-015-1650-6 25804356

[53] Allatif REA Mannaerts GHH Al Afari HST Hammo AN Al Blooshi MS Bekdache OA Concomitant cholecystectomy for asymptomatic gallstones in bariatric surgery-safety profile and feasibility in a large tertiary referral bariatric center. Obes Surg. (2022) 32(2):295–301. 10.1007/s11695-021-05798-9 34791618

[54] Hussain A El-Hasani S. Potential benefits of prophylactic cholecystectomy in patients undergoing bariatric bypass Surgery. Obes Surg. (2016) 26(4):865. 10.1007/s11695-016-2089-0 26843079

[55] Courcoulas AP Daigle CR Arterburn DE. Long term outcomes of metabolic/bariatric surgery in adults. Br Med J. (2023) 383:e071027. 10.1136/bmj-2022-071027 38110235

[56] Yan Z Wei M Zhang T Guo J Yu A Liang Y Vagus nerve preservation for early distal gastric cancer with monitoring and indocyanine green labeling: a randomized clinical trial. JAMA Surg. (2025) 160(1):85–92. 10.1001/jamasurg.2024.5077 39535740 PMC11561724

[57] Haal S Guman MSS Boerlage TCC Acherman YIZ de Brauw LM Bruin S Ursodeoxycholic acid for the prevention of symptomatic gallstone disease after bariatric surgery (UPGRADE): a multicentre, double-blind, randomised, placebo-controlled superiority trial. Lancet Gastroenterol Hepatol. (2021) 6(12):993–1001. 10.1016/S2468-1253(21)00301-0 34715031

[58] Haal S Guman MSS de Brauw LM Schouten R van Veen RN Fockens P Cost-effectiveness of ursodeoxycholic acid in preventing new-onset symptomatic gallstone disease after Roux-en-Y gastric bypass surgery. Br J Surg. (2022) 109(11):1116–23. 10.1093/bjs/znac273 35979609 PMC10364680

[59] Guarino MP Cocca S Altomare A Emerenziani S Cicala M. Ursodeoxycholic acid therapy in gallbladder disease, a story not yet completed. World J Gastroenterol. (2013) 19(31):5029–34. 10.3748/wjg.v19.i31.5029 23964136 PMC3746374

[60] Mulliri A Menahem B Alves A Dupont B. Ursodeoxycholic acid for the prevention of gallstones and subsequent cholecystectomy after bariatric surgery: a meta-analysis of randomized controlled trials. J Gastroenterol. (2022) 57(8):529–39. 10.1007/s00535-022-01886-4 35704084

[61] Fearon NM Kearns EC Kennedy CA Conneely JB Heneghan HM. The impact of ursodeoxycholic acid on gallstone disease after bariatric surgery: a meta-analysis of randomized control trials. Surg Obes Relat Dis. (2022) 18(1):77–84. 10.1016/j.soard.2021.10.004 34772614

[62] Festi D Colecchia A Orsini M Sangermano A Sottili S Simoni P Gallbladder motility and gallstone formation in obese patients following very low calorie diets. Use it (fat) to lose it (well). Int J Obes Relat Metab Disord. (1998) 22(6):592–600. 10.1038/sj.ijo.0800634 9665682

[63] Méndez-Sánchez N González V Aguayo P Sánchez JM Tanimoto MA Elizondo J Fish oil (n-3) polyunsaturated fatty acids beneficially affect biliary cholesterol nucleation time in obese women losing weight. J Nutr. (2001) 131(9):2300–3. 10.1093/jn/131.9.2300 11533270

[64] Damaiyanti DW Tsai ZY Masbuchin AN Huang CY Liu PY. Interplay between fish oil, obesity and cardiometabolic diabetes. J Formos Med Assoc. (2023) 122(7):528–39. 10.1016/j.jfma.2023.03.013 37002172

[65] Wang M Guo J Sun S. Dietary fatty acids and gallstone risk: insights from NHANES and Mendelian randomization analysis. Front Nutr. (2024) 11:1454648. 10.3389/fnut.2024.1454648 39211832 PMC11358065

[66] Kim JK Cho SM Kang SH Kim E Yi H Yun ES N-3 polyunsaturated fatty acid attenuates cholesterol gallstones by suppressing mucin production with a high cholesterol diet in mice. J Gastroenterol Hepatol. (2012) 27(11):1745–51. 10.1111/j.1440-1746.2012.07227.x 22849613

[67] Lee SY Jang SI Cho JH Do MY Lee SY Choi A Gallstone dissolution effects of combination therapy with n-3 polyunsaturated fatty acids and ursodeoxycholic acid: a randomized, prospective, preliminary clinical trial. Gut Liver. (2024) 18(6):1069–79. 10.5009/gnl230494 38712398 PMC11565012

[68] Takeda Y Itoh H Kobashi K. Effect of Clostridium butyricum on the formation and dissolution of gallstones in experimental cholesterol cholelithiasis. Life Sci. (1983) 32(5):541–6. 10.1016/0024-3205(83)90149-2 6823210

[69] Oh JK Kim YR Lee B Choi YM Kim SH. Prevention of cholesterol gallstone formation by *Lactobacillus acidophilus* ATCC 43121 and *Lactobacillus fermentum* MF27 in lithogenic diet-induced mice. Food Sci Anim Resour. (2021) 41(2):343–52. 10.5851/kosfa.2020.e93 33987554 PMC8115012

[70] Han ML Lee MH Lee WJ Chen SC Almalki OM Chen JC Probiotics for gallstone prevention in patients with bariatric surgery: a prospective randomized trial. Asian J Surg. (2022) 45(12):2664–9. 10.1016/j.asjsur.2022.01.120 35232647

[71] Choi SB Lew LC Yeo SK Nair Parvathy S Liong MT. Probiotics and the BSH-related cholesterol lowering mechanism: a jekyll and hyde scenario. Crit Rev Biotechnol. (2015) 35(3):392–401. 10.3109/07388551.2014.889077 24575869

